# Supplementary material for: Learning to assist smokers through encounters with standardized patients: An innovative training for physicians in an Eastern European country
Source: PLoS One. 2019 Sep 26;14(9):e0222813. doi: 10.1371/journal.pone.0222813 (PMC6762076; doi:10.1371/journal.pone.0222813)
Supplement: S1 File — (DOCX) [file pone.0222813.s001.docx]

**SELF-EVALUATION QUESTIONNAIRE**

Please fill a self-assessment form before your training program. This questionnaire aims at evaluation of your experience with counseling of patients and providing an advice to smokers. What strategies do you currently use to help your patients get rid tobacco addiction?

| **Strategy** | **Always** | **Often** | **Sometimes** | **Never** |
| --- | --- | --- | --- | --- |
| I ask if the patient smokes |  |  |  |  |
| I ask about the daily cigarettes number |  |  |  |  |
| I ask about the time of the first cigarette |  |  |  |  |
| I ask if the patient smokes indoor at home |  |  |  |  |
| I ask if the patient intends to stop smoking |  |  |  |  |
| I advise to reduce the number of daily cigarettes |  |  |  |  |
| I advise to stop smoking abruptly |  |  |  |  |
| I discuss the health risks of smoking |  |  |  |  |
| I discuss the benefits of smoking cessation |  |  |  |  |
| I discuss personal barriers to cessation |  |  |  |  |
| I propose to help the patient in quitting |  |  |  |  |
| I advise on behavioral “tricks” |  |  |  |  |
| I give a practical advice to prevent relapse |  |  |  |  |
| I give self-help materials |  |  |  |  |
| I propose an appointment to discuss smoking |  |  |  |  |
| I refer the patient to other specialists (please specify__________________________ ) |  |  |  |  |
| I suggest to set a specific date for quitting smoking |  |  |  |  |
| I prescribe Tabex |  |  |  |  |
| I prescribe NRT (gum) |  |  |  |  |
| I prescribe NRT (patch) |  |  |  |  |
| I prescribe other pharmacological treatment ( please specify _______________________________ ) |  |  |  |  |
| I use another strategy (please specify _______________________________ ) |  |  |  |  |

1. What strategies, in your opinion, will allow for a better and more structured advice to smokers?

__________________________________________________________________________________________________________________________________________________________________________________________________________________________________________________________________________________________________________________________________________________________________________________________________________________________________________________________________

THANK YOU

**ԻՆՔՆԱԳՆԱՀԱՏՄԱՆ ՀԱՐՑԱԹԵՐԹԻԿ**

Դասընթացի սկզբում առաջարկում ենք մի վարժություն, որն ընձեռնում է ծխող հիվանդների խորհրդատվության Ձեր սեփական փորձի նախնական գնահատման հնարավորություն: Ներկայումս ինչպիսի՞ ռազմավարություններ եք կիրառում, որպեսզի օգնեք Ձեր հիվանդներին ազատվել ծխախոտամոլությունից:

|  | **Ռազմավարություն** | **Միշտ** | **Հաճախ** | **Երբեմն** | **Երբեք** |
| --- | --- | --- | --- | --- | --- |
|  | Ես հարցնում եմ, թե հիվանդը ծխում է թե ոչ |  |  |  |  |
|  | Ես հարցնում եմ, թե օրական որքան է ծխախոտ օգտագործում |  |  |  |  |
|  | Ես հարցնում եմ, թե արթնանալուց հետո երբ է ծխում առաջին ծխախոտը |  |  |  |  |
|  | Ես հարցնում եմ, թե արդյոք նա ծխում է տանը փակ տարածքում |  |  |  |  |
|  | Ես հարցնում եմ, թե արդյոք մտադիր է դադարեցնել ծխելը |  |  |  |  |
|  | Ես խորհուրդ են տալիս նվազեցնել ծխախոտի օգտագործումը |  |  |  |  |
|  | Ես խորհուրդ եմ տալիս ամբողջովին դադարեցնել ծխելը |  |  |  |  |
|  | Ես քննարկում եմ առողջության համար ծխելու վտանգները |  |  |  |  |
|  | Ես քննարկում եմ ծխելու դադարեցման առավելությունները |  |  |  |  |
|  | Ես քննարկում եմ ծխելը թողնելու անհատական ​​խոչընդոտները |  |  |  |  |
|  | Առաջարկում եմ օգնել հիվանդին ծխելը դադարեցնելու հարցում |  |  |  |  |
|  | Վարքագծի փոփոխության «հնարքներ» եմ առաջարկում |  |  |  |  |
|  | Ես տալիս եմ գործնական խորհուրդներ` ձախողումը կանխելու նպատակով |  |  |  |  |
|  | **Ռազմավարություն** | **Միշտ** | **Հաճախ** | **Երբեմն** | **Երբեք** |
|  | Ես առաջարկում եմ տեղեկատվական նյութեր |  |  |  |  |
|  | Առաջարկում եմ նշանակել հանդիպում` ծխելը դադարեցնելու մասին խոսելու համար |  |  |  |  |
|  | Ես խորհուրդ եմ տալիս այդ հարցով դիմել այլ մասնագետների օգնությանը (խնդրում ենք նշել ինչպիսի ……………………………………..) |  |  |  |  |
|  | Ես առաջարկում եմ սահմանել կոնկրետ օր` ծխելը դադարեցնելու համար |  |  |  |  |
|  | Ես նշանակում եմ տաբեքս  (Tabex) |  |  |  |  |
|  | Ես նշանակում եմ նիկոտին պարունակող մաստակ |  |  |  |  |
|  | Ես նշանակում եմ նիկոտին պարունակող սպեղանի |  |  |  |  |
|  | Ես նշանակում եմ այլ դեղամիջոց (խնդրում ենք նշել ինչպիսի … ..……………………………) |  |  |  |  |
|  | Ես օգտագործում եմ մեկ այլ ռազմավարություն (խնդրում ենք նշել ինչպիսի……………………………………………) |  |  |  |  |

Որոնք են, Ձեր կարծիքով, այն ռազմավարությունները, որ թույլ կտան ավելի լավ և համակարգված օժանդակել Ձեր հիվանդներին ծխելը թողնելու հարցում:

……………………………………………………………………………………………………………….………………………………………………………………………………………………………………….………………………………………………………………………………………………………………….………………………………………………………………………………………………………………….………………………………………….………………………………………………………………………………………………………………………………………………………………………………………….…………………...……………………………………………………………………………………………..…………………………………………………………………………………………………………………….

ՇՆՈՐՀԱԿԱԼՈՒԹՅՈՒՆ
