# Supplementary material for: Learning to assist smokers through encounters with standardized patients: An innovative training for physicians in an Eastern European country
Source: PLoS One. 2019 Sep 26;14(9):e0222813. doi: 10.1371/journal.pone.0222813 (PMC6762076; doi:10.1371/journal.pone.0222813)
Supplement: S2 File — (DOCX) [file pone.0222813.s002.docx]

**Training evaluation questionnaire**

1. Please indicate 3 main positive points of the training course: ………………………………………………………………………………………………………………………………………………………………………………………………………………………………………………………………………………………………………………………………………………….……………………………………………………………………………………………………………………………………………………………………………………………………………………………………………………………………………………………………………………………………………………………………………………………………………………………………………………………………………………………………………………………………………………………………………
2. Please indicate 3 main points in the course that could be improved:

………………………………………………………………………………………………………………………………………………………………………………………………………………………………………………………………………………………………………………………………………………….………………………………………………………………………………………………………………………………………………………………………………………………………………………………………………………………………………………………………………………………………………………………………………………………………………………

……………………………………………………………………………………………………………………………………………………

1. To what extent did the course succeed in achieving its objectives such as:

| Objective | Not achieved at all | Partially achieved | Completely achieved |
| --- | --- | --- | --- |
| To know the risks of tobacco use |  |  |  |
| To know the benefits of smoking cessation |  |  |  |
| To understand the tobacco dependence |  |  |  |
| To know the stages of the behavior change |  |  |  |
| To understand the physician’s role in smoking cessation |  |  |  |
| To advise smokers with strategies matching their motivation to stop |  |  |  |
| To prescribe pharmacological therapy for smoking cessation |  |  |  |
| To increase your self-confidence in helping the patients to fight the tobacco addiction |  |  |  |
| To build your skills for intervening the patients who smoke |  |  |  |
| To increase your self-efficacy in assisting your patients to stop smoking |  |  |  |

1. Please rate the duration of the course:

|  | Too short | Somewhat short | Right duration | Somewhat long | Too long |
| --- | --- | --- | --- | --- | --- |
| The course overall |  |  |  |  |  |
| Video-material |  |  |  |  |  |
| Discussions |  |  |  |  |  |
| Role plays |  |  |  |  |  |
| Practice with standardized patients |  |  |  |  |  |

1. Please assess the overall quality of the course:

| Statement | Completely disagree | Disagree | Neutral | Somewhat agree | Completely agree |
| --- | --- | --- | --- | --- | --- |
| The objectives of the course were clearly stated |  |  |  |  |  |
| I learned a lot during the course |  |  |  |  |  |
| I can apply the in practice what I learned |  |  |  |  |  |
| In general, the course was well organized |  |  |  |  |  |
| The course materials were helpful |  |  |  |  |  |
| The atmosphere during the course was pleasant |  |  |  |  |  |
| I am satisfied with the course |  |  |  |  |  |
| I would recommend this course to my colleagues |  |  |  |  |  |

**THANK YOU**

**Դասընթացի գնահատման**

**ՀԱՐՑԱԹԵՐԹԻԿ**

1. **Խնդրում ենք նշել դասընթացի 3 հիմնական դրական կողմերը։**

……………………………………………………………………………………………………………………………………………………………………………………………………………………………………………………………………………………………………………………………………………………………………………………………………………………………………………………………………………………………………………………………………………………………………………………………………………………………………………………………………………………………………………………………………………………………………………………………………………………………………………………

1. **Խնդրում ենք նշել դասընթացում առկա 3 հիմնական թերությունները։**

……………………………………………………………………………………………………………………………………………………………………………………………………………………………………………………………………………………………………………………………………………………………………………………………………………………………………………………………………………………………………………………………………………………………………………………………………………………………………………………………………………………………………………………………………………………………………………………………………………………………………………………

1. **Որքանո՞վ է դասընթացը հաջողել իր նպատակներին հասնելու հարցում։**

| Հարցադրում | Բոլորովին չի հաջողել | Մասամբ հաջողել է | Լիովին հաջողել է |
| --- | --- | --- | --- |
| Իմանալ ծխելու ռիսկերը |  |  |  |
| Իմանալ ծխելը դադարեցնելու առավելությունները |  |  |  |
| Հասկանալ նիկոտինային կախվածությունը |  |  |  |
| Հասկանալ վարքագծի փոփոխության փուլերը |  |  |  |
| Պատկերացնել բժշկի դերը ծխելը դադարեցնելու գործընթացում |  |  |  |
| Տրամադրել ծխելը դադարեցնելուն ուղղված խորհրդատվություն համաձայն հիվանդի մոտիվացիոն փուլի |  |  |  |
| Նշանակել դեղամիջոցներ` ծխելը դադարեցնելուն օժանդակելու նպատակով |  |  |  |
| Բարձրացնել սեփական ուժերի նկատմամբ Ձեր վստահությունը հիվանդին ծխախոտամոլությունից ազատելու հարցում |  |  |  |
| Ձևավորել և վարժել հիվանդի ծխելու հարցում միջամտելու Ձեր հմտությունները |  |  |  |
| Բարձրացնել հիվանդին ծխելը դադարեցնելու հարցում օգնելու Ձեր հաստատակամությունը և ունակությունը |  |  |  |

1. **Խնդրում ենք գնահատել դասընթացի տևողությունը։**

| Հարցադրում | Շատ կարճ | Փոքր-ինչ կարճ | Ոչ կարճ, ոչ երկար | Փոքր-ինչ երկար | Շատ երկար |
| --- | --- | --- | --- | --- | --- |
| Դասընթացը ընդհանուր առմամբ |  |  |  |  |  |
| Տեսանյութերը |  |  |  |  |  |
| Քննարկումները |  |  |  |  |  |
| Դերախաղերը |  |  |  |  |  |
| Վարժանքներ ստ. պացիենտների հետ |  |  |  |  |  |

1. **Խնդրում ենք գնահատել դասընթացը ընդհանուր առմամբ։**

| Հարցադրում | Բոլորովին համաձայն չեմ | Համաձայն չեմ | Ոչ համաձայն եմ, ոչ էլ չեմ | Որոշ չափով համաձայն եմ | Լիովին համաձայն եմ |
| --- | --- | --- | --- | --- | --- |
| Դասընթացի նպատակները  ճշգրիտ կերպով պարզաբանվել են |  |  |  |  |  |
| Ես շատ բան եմ սովորել |  |  |  |  |  |
| Ես կարող եմ կիրառել սովորածս գործնականում |  |  |  |  |  |
| Ընդհանուր առմամբ, դասընթացը լավ էր կազմակերպված |  |  |  |  |  |
| Դասընթացի նյութերն օգտակար էին |  |  |  |  |  |
| Դասընթացի ընթացքում հաճելի մթնոլորտ էր |  |  |  |  |  |
| Ես գոհ եմ դասընթացից |  |  |  |  |  |
| Ես խորհուրդ կտայի այս դասընթացն իմ գործընկերներին |  |  |  |  |  |

ՇՆՈՐՀԱԿԱԼՈՒԹՅՈՒՆ
