# Supplementary material for: Learning to assist smokers through encounters with standardized patients: An innovative training for physicians in an Eastern European country
Source: PLoS One. 2019 Sep 26;14(9):e0222813. doi: 10.1371/journal.pone.0222813 (PMC6762076; doi:10.1371/journal.pone.0222813)
Supplement: Attachment — Submitted filename: Response to In-House-Review_June 17 2019.docx [file pone.0222813.s003.docx]

EDITORIAL TEAM

PLOS ONE

PONE-D-19-16171
Learning to assist smokers through encounters with standardized patients: an innovative training for physicians in an Eastern European country

June 17, 2019

Dear Samantha Russell,

Thank you for your feedback on the manuscript entitled "Learning to assist smokers through encounters with standardized patients: an innovative training for physicians in an Eastern European country" submitted to PLOS ONE.

I am pleased to provide additional details and supporting information to address the identified issues as suggested in your letter.

In particular, the Materials and Methods section has been reorganized in 7 sub-sections (Study setting, Data collection and instruments, Analysis, Recruitment, Training materials, Standardized patient training, and Training) to provide a more detailed description of the study methodology. This includes new details about the study settings, the adaptation of the training materials, the design of the questionnaires and the participants’ recruitment process as following (the added text is in blue color):

**“Study setting**

This training course was conducted at the American University of Armenia (AUA) in Yerevan. The study participants were medical residents of Yerevan State Medical University (YSMU).

**Data collection and instruments**

Data for this study were collected from medical residents from May 2015 to January 2016.

Data collection included an evaluation survey at the completion of the training course, and a self-assessment of smoking cessation counselling skills before and 6 months after the training course, to allow participants time to practice the skills acquired. All surveys were self-administered, anonymous, and collected by a third party in the absence of the course instructor. The questionnaires validated in previous studies (7) were translated from English in Armenian. The questionnaire that evaluated the cessation counselling course was administered immediately after the training and included 10 items on the course objectives (three-point rating scale), five items to assess the course duration and seven items on the overall quality of the course (five-point Likert scale). The respondents also answered two open-ended questions regarding the strengths and weaknesses of the training course.

The questionnaire that evaluated the medical residents’ smoking cessation counselling activities contained 23 items covering tobacco dependence assessment, smoking cessation counselling strategies, and pharmacological treatment. The structured questionnaire had four-point Likert-type response options to assess the residents’ use of counselling strategies before and 6 months after the training, including the “5 As”: asking about tobacco use, advising every smoker to quit, assessing their willingness to quit, assisting in making a quit attempt and arranging a follow-up visit (8), among others.

**Analysis**

We assumed that a sample size of 40 should suffice for this feasibility study. A non-parametric Mann-Whitney test was used to assess pre-post changes in physicians’ counselling skills measured on an ordinal scale (Likert-type questions). Descriptive statistics were calculated for the overall assessment of the course. Statistical significance was defined at p<0.05.

**Recruitment**

The Faculty of Postgraduate and Continuing Medical Education contacted in May-June 2015 a selected sample of medical residents in different years of study at Yerevan State Medical University and invited to the training. The inclusion criteria were the specialization in cardiology, gastroenterology and pulmonology as it was assumed that these specialists were more likely to devote time to smoking cessation counselling. Residents in surgery, gynaecology, emergency medicine and other narrow specialties were not invited. No other restrictions were applied in the recruitment process. All residents meeting the inclusion criteria were invited to the training.

**Training materials**

The original Swiss materials, including trainer’s manual, case scenarios, slides, video-taped counselling sessions, and patient brochures were translated from French into Armenian by professional services. The Armenian lead co-investigator (first author) reviewed the translated materials for the medical terminology and the relevance to local context and added the data on smoking epidemics in Armenia. In addition, two teaching faculty members of the YSMU Department of Family Medicine reviewed and adjusted the case scenarios and the patient brochures for cultural appropriateness.”

Next, the information on the participants’ demographic and other characteristics is now available in the Table 1 (newly added). The study participants’ sample cannot be considered as representative which is stated now in the Discussion section as a limitation:

“Besides, the participants represented only selected fields of specialization whereas other specialists may have a different level of interest in smoking cessation counselling training”.

The subsection on the statistical analysis has been slightly expanded and more details on the study results can be found in the Table 2 (newly added).

Finally, we provide the English version of the study questionnaires that were validated in previous studies, along with the Armenian translated versions, as Supportive Information, as suggested.

We sincerely hope these additions do address your questions and concerns and may help the review process.

Thank you very much for your consideration.

Sincerely,

Narine Movsisyan, MD, MPH

Senior Researcher

School of Public Health

American University of Armenia

40 Baghramyan Avenue

Yerevan 0019 Armenia

Phone: +374 60 612 592
