# Supplementary material for: Learning to assist smokers through encounters with standardized patients: An innovative training for physicians in an Eastern European country
Source: PLoS One. 2019 Sep 26;14(9):e0222813. doi: 10.1371/journal.pone.0222813 (PMC6762076; doi:10.1371/journal.pone.0222813)
Supplement: Attachment — Submitted filename: Response to Reviewers_August 19 2019.docx [file pone.0222813.s004.docx]

August 19, 2019

**Dr. Conor Gilligan**
Academic Editor
PLOS ONE

PONE-D-19-16171
Learning to assist smokers through encounters with standardized patients: an innovative training for physicians in an Eastern European country

Dear Dr. Gilligan,

Thank you very much for the review of our manuscript "Learning to assist smokers through encounters with standardized patients: an innovative training for physicians in an Eastern European country" and the valuable feedback.

On behalf of the authors’ team, I am pleased to submit a revised manuscript with addressed comments raised by the editorial team during the review process. Please kindly find the point-by-point description of our responses to the editors’ and reviewer’s comments.

Thank you very much for your consideration.

Sincerely,

Narine Movsisyan, MD, MPH

Senior Researcher

School of Public Health

American University of Armenia

40 Baghramyan Avenue

Yerevan 0019 Armenia

Phone: +374 60 612 592

**Responses to the Editor’s comments**

**Editor’s comments:**

1. Please check your reference formatting – need a space before the (citation).

**Response:** The formatting was corrected throughout the text.

2. Page 3 ‘one such scheme’ – it is unclear what this refers to. I suggest breaking the introduction into several paragraphs and beginning a new one at this point – introduce the concept of various programs designed to improve physicians smoking cessation skills and provide a few examples of other programs – not just the Swiss example you have adapted.

**Response:** Thank you very much for the suggestion. We started a new paragraph with more information on the smoking cessation graduate training programs available in Europe to put the Swiss program into the context.

*“Many postgraduate programs for physicians’ smoking cessation training are available in Europe; however, their effectiveness in changing provider practices and patient smoking outcomes is not adequately evaluated. Most often, these courses cover brief intervention, pharmacotherapy, motivational interviewing skills and the stages of behavioural change (7).* One such scheme, an innovative postgraduate training program implemented in teaching hospitals in Switzerland, was shown to improve both physician counselling skills and patient-related outcomes (3).*”*

3. Please clarify and emphasise this as a feasibility study and note the future need to evaluate effectiveness through evaluation of practitioner behaviour.

**Response:** We now clearly state that this is a feasibility study and that future studies would need to employ a more rigorous design for the effectiveness evaluation.

*“No control group was used in this feasibility study with a pre-post design; therefore, the effectiveness of the training program cannot be determined.*

Future studies will aim *to evaluate the effectiveness of the training model in changing provider and patient related outcomes* and to explore the ways to further improve physician counselling and prescribing skills to reach out to the smoker patients in restricted-resource settings.”

4. Please correct your reference to "p=0.000" to "p<0.001" or as similarly appropriate, as p values cannot equal zero.

**Response:** Done.

**Responses to the Reviewer’s comments**

**Reviewer’s comments:**

The revised version of this manuscript addresses the previous issues raised. Below are some minor additional suggestions.

1. In the discussion could consider acknowledging that the design was a pre-post design for this feasibility study and given that there was no control group the effectiveness of the training program cannot be determined.

**Response:** Thank you for your suggestion. We have added a clear statement on this important study limitation.

*“No control group was used in this feasibility study with a pre-post design; therefore, the effectiveness of the training program cannot be determined”.*

2. In the analysis section could consider rephrasing the first sentence to "Given the purpose of this study was to examine feasibility we aimed to recruit a sample size of 40 participants."

**Response:** The justification for the study sample size was re-phrased as suggested.

*“Given the purpose of this study was to examine the feasibility of the intervention we aimed to recruit a sample size of 40 participants.”*
